# Validation of a yeast malate dehydrogenase 2 (Mdh2) antibody tested for use in western blots

**DOI:** 10.12688/f1000research.13396.2

**Published:** 2018-07-30

**Authors:** Shiran Gabay-Maskit, Maya Schuldiner, Einat Zalckvar

**Affiliations:** 1 Department of Molecular Genetics, Weizmann Institute of Science, Rehovot, 7610001, Israel

**Keywords:** Malate dehydrogenase, Saccharomyces cerevisiae, antibody, western blot

## Abstract

Malate dehydrogenases (Mdhs) reversibly convert malate to oxaloacetate and serve as important enzymes in several metabolic pathways. In the yeast *Saccharomyces cerevisiae* there are three Mdh isozymes, localized to different compartments in the cell. In order to identify specifically the Mdh2 isozyme, GenScript USA produced three different antibodies that we further tested by western blot. All three antibodies recognized the *S. cerevisiae* Mdh2 with different background and specificity properties. One of the antibodies had a relatively low background and high specificity and thus can be used for specific identification of Mdh2 in various experimental settings.

## Introduction

Malate dehydrogenases (Mdhs) catalyse the interconversion of malate and oxaloacetate using NAD ^+^ or NADH ^1^. In the yeast *Saccharomyces cerevisiae* there are three known isozymes: Mdh1 is located in mitochondria, Mdh2 is mostly cytosolic, and Mdh3 is localized to peroxisomes. The residues of the three isozymes are between 43–50% identical ^2^. It is thus very important for purposes of isolation and identification to utilize specific antibodies that will recognize only one specific isozyme. Using three Mdh2 peptides, which were specifically designed to unique regions in the Mdh2 protein, GenScript USA produced three different antibodies that should have high specificity for *S. cerevisiae* Mdh2 relative to Mdh1 and Mdh3. We then tested all three antibodies by western blotting and found one with specific binding. Due to its specificity, this antibody has the potential to also work in other experimental assays such as immunoprecipitation, immunohistochemistry and ELISA (Enzyme-Linked Immunosorbent Assay).

## Methods

### Antibody details

Three antibodies for *S. cerevisae* Mdh2 were custom produced for us by GenScript USA : 1. Purified antibody, Anti-peptide #1, item number: U1684BK300_2, LOT number: A416120074, catalogue number: SC1195. 2. Purified antibody, Anti-peptide #2, item number: U1684BK300_5, LOT number: A416120094, catalogue number: SC1195. 3. Purified antibody, Anti-peptide #3, item number: U1684BK300_8, LOT number: A416120072, catalogue number: SC1195.

All three antibodies were raised in New Zealand Rabbit and are polyclonal. The immunogen for all three antibodies is conjugated KLH Peptide. To find specific Mdh2 peptides, the three *S. cerevisiae* malate dehydrogenases (Mdh1, Mdh2 and Mdh3) were aligned and analysed by GenScript, using their Antigen Design Tool. From this analysis, three peptides (corresponding to the three antibodies) were selected: #1: CHPQSRNSGIERRIM; #2: CINIESGLTPRVNSM; #3: MPHSVTPSIEQDSLC. The cysteines in the N’ terminus (peptides number 1 and 2) or C’ terminus (peptide number 3) were added for KLH conjugation. 

### Yeast strains and strain construction

Yeast strains are all based on the BY4741 laboratory strain ^3^. Manipulations were performed using a standard PEG/LiAC transformation protocol ^4^. The GFP-Mdh1, GFP-Mdh2, GFP-Mdh3 and OE-mCherry-Mdh3 strains were picked from the SWAT N’ GFP or N’ mCherry yeast libraries that were recently prepared in our lab ^5^. All Strains with a fluorescent tag, including the strains that were picked from the SWAT libraries were verified using PCR (one primer from within the endogenous Open Reading Frame (ORF) and one from within the tag sequence) as well as by fluorescent microscopy. Primers for creating strains with deletions or C’ tagging of genes (Δ *mdh1*, Δ *mdh2*, Δ *mdh3* and Mdh2-mCherry) were designed using the Primers-4-Yeast web tool ^6^. All deletions were verified using primers from within the endogenous ORF. All primers are summarized in Table 1.

**Table 1.** A list of primers used in this study.

Gene	Primer Name	Sequence	Description
*MDH1*	*MDH1* KO pFA6 F	AAAAAAAACAAAAGGAAAAGGAAGGATACCATATACAATGCGGATCCCCGGGTTAATTAA	Primer for KO of gene using pFA6 plasmids.
*MDH1*	*MDH1* KO pFA6 R	TTCCCTATTTTTCACTCTATTTCTGATCTTGAACAATCTAGAATTCGAGCTCGTTTAAAC	Primer for KO of gene using pFA6 plasmids.
*MDH1*	*MDH1* N' tag CHK R	TGGAACGGTAGAATTGACTG	Primer for checking N' tagging of the gene.
*MDH1*	*MDH1* WT CHK F	TCCAACCCAGTCAATTCTAC	Primer for testing the presence of the gene.
*MDH1*	*MDH1* WT CHK R	GTTAGCGAATTTAGCACCAG	Primer for testing the presence of the gene.
*MDH2*	*MDH2* KO pFA6 F	CAAAAGTTCAATACAATATCATAAAAGTTATAGTAACATGCGGATCCCCGGGTTAATTAA	Primer for KO of gene using pFA6 plasmids.
*MDH2*	*MDH2* KO pFA6 R	CAATTTGCTGCATTCTTATGCTTCGGTCCGATGCTCATTAGAATTCGAGCTCGTTTAAAC	Primer for KO of gene using pFA6 plasmids.
*MDH2*	*MDH2* N' tag CHK R	TGGAAATGACAAGAACGAAG	Primer for checking N' tagging of the gene.
*MDH2*	*MDH2* WT CHK F	AACTGTTTGCATAACGCTTC	Primer for testing the presence of the gene.
*MDH2*	*MDH2* WT CHK R	CATGGAGTTAACACGAGGAG	Primer for testing the presence of the gene.
*MDH2*	*MDH2* C' tag CHK F	ATTCCGTTGTTTTCACAGTC	Primer for checking C' tagging of the gene.
*MDH2*	*MDH2* C' tag pFA6 F	TAAGGGCTTGGAATTCGTTGCATCGAGATCTGCATCATCTCGGATCCCCGGGTTAATTAA	Primer for C' tagging of gene using pFA6 plasmids.
*MDH2*	*MDH2* C' tag pFA6 R	CAATTTGCTGCATTCTTATGCTTCGGTCCGATGCTCATTAGAATTCGAGCTCGTTTAAAC	Primer for C' tagging of gene using pFA6 plasmids.
*MDH3*	*MDH3* KO pFA6 F	TGCAAAAGAAAATAAAAAGAGACAAACAATCATAAACATGCGGATCCCCGGGTTAATTAA	Primer for KO of gene using pFA6 plasmids.
*MDH3*	*MDH3* KO pFA6 R	AGTATAGAGTTAAGAAAAATATAAAAATTGAAGTAGCTCAGAATTCGAGCTCGTTTAAAC	Primer for KO of gene using pFA6 plasmids.
*MDH3*	*MDH3* N' tag CHK R	TTCTTCAAAGTTTCCACAGC	Primer for checking N' tagging of the gene.
*MDH3*	*MDH3* WT CHK F	ATTCAGGGGAAACCATTATC	Primer for testing the presence of the gene.
*MDH3*	*MDH3* WT CHK R	TCGATGGATACTACGCTACC	Primer for testing the presence of the gene.
*MDH1/2/3*	N 'tag CHK F	CGACAGAGAATTCATCGATG	Primer for checking N' tagging. Using pym plasmid
*MDH1/2/3*	F2-Rev-Com	TTAATTAACCCGGGGATCCG	Reverse complementary to the F1/F2 primer of the Longtine's pFA6a plasmids.

### Western blot analysis

Yeast proteins were extracted by the NaOH protocol as previously described ^7^ and resolved on polyacrylamide gels with the following modifications:

1. Liquid cultures were grown over night at 30°C in glucose containing media, and then were diluted and incubated for an additional 4–6 hours to get to mid-log phase. Cells that were grown in a different carbon source than glucose were grown in synthetic media with 0.1% glucose (6.7 g/l yeast nitrogen base and 0.1% glucose) for the overnight incubation, then diluted in synthetic media containing galactose (6.7 g/l yeast nitrogen base and 2% galactose) or oleic acid (6.7 g/l yeast nitrogen base, 0.2% oleic acid and 0.1% Tween-80) and incubated for an additional 4–6 hours. Cells that were grown in glucose were grown in yeast extract peptone dextrose (YPD) rich medium (1% Yeast Extract, 2% peptone, 2% glucose) for the whole process.
2. The extractions were incubated for 10 minutes at 70°C and 15 µl supernatant was loaded per lane of the gel.

The gels were transferred to nitrocellulose membrane blots, blocked for 1 hour in SEA block diluted in TBS-T (1:5) at room temperature (RT), and probed with primary rabbit/mouse antibodies against Mdh2, Histone H3, Actin and mCherry ( Table 2) for 1 hour at RT. Final concentrations of the primary antibodies were: 1 µg/ml for anti-Mdh2 peptides #1 and #3, 0.5 µg/ml for anti-Mdh2 peptide #2, anti-mCherry and anti-Actin and 0.2 µg/ml for anti-Histone H3. The membranes were washed 3 times without incubation in TBS-T, followed by 3x3 minute washes in TBS-T. The membranes were then probed with a secondary goat anti-rabbit/mouse antibody conjugated to IRDye800 or to IRDye680 ( Table 2) for 30 minutes at RT and washed as before. Final concentrations of the secondary antibodies were 0.1 µg/ml for IRDye 800CW Goat anti-Rabbit IgG and IRDye 680RD Goat anti-mouse IgG, and 0.2 µg/ml for Goat anti-mouse HRP conjugated. Membranes were scanned for infrared signal using the Odyssey Imaging System (Li-Cor). For detecting the anti-Actin primary antibody ( Table 2), a goat anti-mouse HRP conjugated secondary antibody ( Table 2) was used. The membrane was then washed with ECL reagents and scanned using imageQuant LAS 4000 system (GE Healthcare). Images were transferred to ImageJ 1.51s for slight adjustments of contrast and brightness. For further information about the western blot reagents see Table 3.

**Table 2.** Primary and secondary antibodies.

Antibody	Manufacturer	Catalogue Number	RRID
Rabbit anti-Mdh2 peptide #1 (Ab1)	GenScript	SC1195	
Rabbit anti-Mdh2 peptide #2 (Ab2)	GenScript	SC1195	
Rabbit anti-Mdh2 peptide #3 (Ab3)	GenScript	SC1195	
Mouse anti-mCherry	Abcam	ab125096	AB_11133266
Rabbit anti-Histone H3	Abcam	ab1791	AB_302613
Mouse anti-Actin	Abcam	ab8224	AB_449644
IRDye 800CW Goat anti-Rabbit IgG	LI-COR, Inc	926-32211	AB_621843
IRDye 680RD Goat anti-mouse IgG	LI-COR, Inc	926-68070	AB_10956588
Goat anti-mouse HRP conjugated	Abcam	ab6789	AB_955439

**Table 3.** Details of reagents for Western Blot.

Process	Reagent	Manufacturer	Catalogue Number	Concentration
Protein extraction	Sodium hydroxide (NaOH)	Merck Chemicals	1064981000	0.2 M
5 X SDS sample buffer	β-mercapto-ethanol	Sigma	02390-25ML	5%
	Bromophenol Blue	Amresco	0449-50G	0.5%
	Tris pH 6.8	MP Biomedicals	819620	0.312 M
	Glycerol	J.T.Baker	2136-01	25%
	SDS	Sigma	L4509-500G	10%
Mdh2 Antibodies Diluent	BSA (in wash buffer)	MP Biomedicals	160069	1%
Other antibodies Diluent	Skim milk powder (in wash buffer)	Tnuva-423	48703	5%
10 X TBS-T Wash buffer	Tris 1 M pH 7.9	MP Biomedicals	819620	10%
	Tween 20	Sigma	P1379-500ML	0.5%
	0.5 M EDTA pH 8.0	J.T.BAKER	8993-01	0.5%
	NaCl	J.T.BAKER	0277	1.5 M
Blocking	SEA block (in wash buffer)	Thermo scientific	37527	20%
Chemiluminescence	Amersham ECL western blot reagents	GE Healthcare	RPN2106	Proprietary

### Controls

Several controls were used in this study. Loading controls for the total amount of protein were performed using anti-Histone H3 or anti-Actin antibodies. A Δ *mdh2* strain, alongside Δ *mdh1* and Δ *mdh3* strains, were used to verify the specificity of the Mdh2 antibodies. An anti-mCherry antibody was used as a control for the bands detected in Mdh2-mCherry or Tef2-mCherry-Mdh3 (over expression) compared to the bands detected by the anti-Mdh2 antibodies. An anti-GFP antibody was used as a control for the bands detected in GFP-Mdh1, GFP-Mdh2 and GFP-Mdh3 with anti Mdh2 antibody peptide #3.

## Results

### The three GenScript antibodies detect Mdh2 from cells grown on oleic acid

For first examination of the three antibodies, we grew control cells in three different carbon sources: glucose, galactose and oleic acid. Since transcription of *S. cerevisiae* Mdh2 is repressed by glucose ^8^, we hypothesized that the antibodies will not detect Mdh2 in extracts of cells grown in glucose, but will detect it in the galactose and oleic acid samples. We then performed protein extraction using the NaOH approach, and examined the three GenScript anti-Mdh2 antibodies.

**Figure 1.**
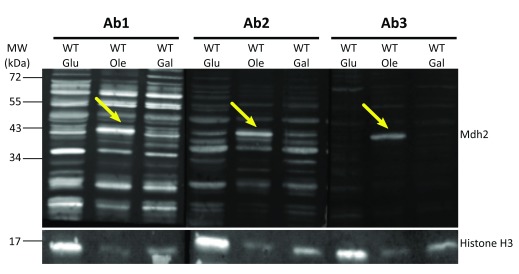
Three new antibodies recognize Mdh2 in cells grown in oleic acid. Western blot analysis was performed on protein extracts of control cells (BY4741) grown in glucose (Glu), oleic acid (Ole) or galactose (Gal) using antibodies 1–3 (Ab1, Ab2, Ab3). Native Mdh2 (41 KDa) is detected by all three anti-Mdh2 antibodies in protein extractions from cells grown in oleic acid as indicated by yellow arrows. Histone H3 was used as a loading control. In total we detected seven times a band at the size of Mdh2 when cells were grown on Oleate: One time using antibody1, three times using antibody 2 and three times using antibody 3.

Indeed, all three antibodies could not detect a specific band at the correct size of Mdh2 (41 KDa) in protein extractions from cells grown in glucose ( Figure 1, Glu). Although the general protein levels of the control strain grown in oleic acid were much lower than from the levels of controls grown in glucose, a prominent band at the correct size for Mdh2 was detected by all three antibodies. In galactose there was no prominent band at the correct size, although this could be due to the general low levels of protein in this condition, as can be seen by the Histone H3 loading control. Endogenous Mdh2 was also recognized in protein extractions from cells grown on other carbon sources such as EtOH (not shown). This suggests that the upregulation of Mdh2 in cells grown in oleic acid is due to the removal of the glucose repression and not because of an oleic acid-specific up-regulation.

### Antibody 3 has high specificity for Mdh2

**Figure 2.**
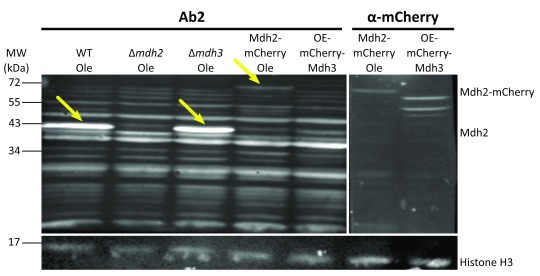
Antibody 2 displays isoform specificity. Western blot analysis was performed on protein extractions from different strains grown on oleic acid (Ole) or in glucose (only for OE-mCherry-Mdh3) using antibody 2 and anti-mCherry antibody. Antibody 2 recognizes the native Mdh2 in control cells and in Δ *mdh3* cells grown in oleic acid, but does not recognize it in Δ *mdh2* strain (second lane). The antibody also recognizes Mdh2 tagged with mCherry ( ^∼^70 kDa), but not the over-expressed and mCherry tagged Mdh3. The last one can be recognized in the control with the anti-mCherry. Histone H3 was used as a loading control. Yellow arrows indicate bands corresponding to Mdh2. (OE = over expression). We saw that antibody 2 specifically recognized Mdh2 and not Mdh3 in three experiments.

**Figure 3.**
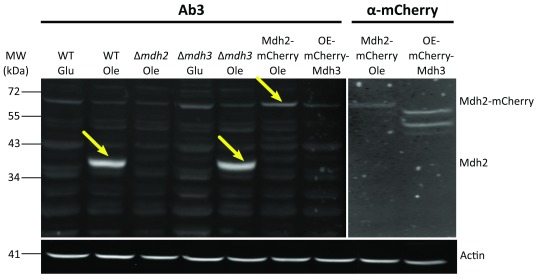
Antibody 3 has the best specificity to Mdh2. Western blot analysis was done on protein extractions from different strains grown on oleic acid (Ole) or glucose (Glu) (OE-mCherry-Mdh3 was grown in glucose) using antibody 3 and anti-mCherry. Similarly to antibody 2, antibody 3 does not recognize Mdh2 in Δ *mdh2* strain or in cells grown on glucose. This antibody has a good specificity to Mdh2, as seen by the low background signal. Actin was used as a loading control. Yellow arrows indicate bands corresponding to Mdh2. (OE = over expression). We saw that antibody 3 specifically recognizes Mdh2 in three experiments.

**Figure 4.**
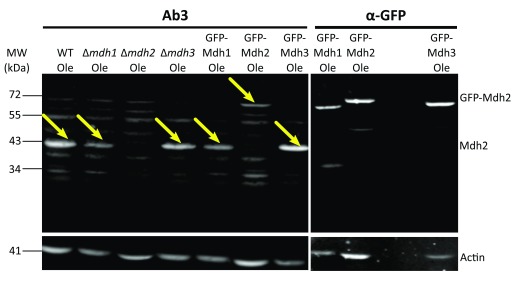
Antibody 3 recognizes Mdh2 in Δ *mdh1* and Δ *mdh3* cells extractions. Western blot analysis was done on protein extractions from different strains grown on oleic acid (Ole) using antibody 3 and an anti-GFP antibody. As shown in Figure 3, this antibody does not recognize Mdh2 in Δ *mdh2* strain or in cells grown on glucose but does recognize Mdh2 in Δ *mdh3* cells grown on oleic acid. Here we show that antibody 3 also recognizes Mdh2 in Δ *mdh1* cells grown on oleic acid. Actin was used as a loading control. Yellow arrows indicate bands corresponding to Mdh2. We saw that antibody 3 specifically recognizes Mdh2 and not Mdh3 in two experiments and that antibody 3 does not recognize Mdh1 in one experiment.

After the first comparison, we focused on antibodies 2 and 3 as they displayed the lowest background signal. We verified the affinity and specificity of the antibodies using several strains, in addition to the control strain: Δ *mdh1,* Δ *mdh2,* Δ *mdh3,* as well as strains that had a mCherry tag fused to either Mdh2 or Mdh3 or GFP tag fused to Mdh1, Mdh2 or Mdh3. Both antibody 2 and antibody 3 detected endogenous Mdh2 in wild type cells and in Δ *mdh3* cells grown in oleic acid ( Figure 2 and Figure 3), but were specific to Mdh2 as they did not cross react in the Δ *mdh2* strain. Another way to demonstrate specificity is to tag Mdh2 with mCherry, thus shifting only this specific isoform in size. Indeed, under these conditions both antibodies 2 and 3 detected a higher protein form only (Mdh2 tagged with mCherry = ^∼^70 kDa), though with lower affinity. Reciprocally, neither antibody detected over expressed mCherry-Mdh3 (expressed under a Tef2 promotor), although it was highly expressed as could be verified by an anti-mCherry antibody. In addition, antibody 3 detected endogenous Mdh2 in Δ *mdh1* cells grown in oleic acid, as well as a bigger band size only in GFP-Mdh2 tagged cells (Mdh2 tagged with GFP = ^∼^68 kDa) ( Figure 4). Although both antibodies fit the requirement of specific identification of the yeast Mdh2, we have decided to use antibody 3, as it has a better specificity as seen from the lower background signal ( Figure 3).

Raw images of all western blots included in figures presented Click here for additional data file. Copyright: © 2018 Gabay-Maskit S et al. 2018 Data associated with the article are available under the terms of the Creative Commons Zero "No rights reserved" data waiver (CC0 1.0 Public domain dedication).

## Conclusion

Three antibodies against endogenous *S. cerevisiae* Mdh2 were prepared by GenScript, using specific peptides of Mdh2 as the immunogens. The antibodies were checked by western blot and were shown to have the ability to detect endogenous Mdh2, when cells are grown under conditions in which Mdh2 is expressed. Although two of the three antibodies demonstrated the specificity qualities required from such an antibody, antibody 3 had the best specificity qualities, and the lowest background signal. We thus recommend antibody 3 as the best option to detect endogenous *S. cerevisiae* Mdh2. This antibody can be used in western blot, as shown in this manuscript, and should be tested for other uses (e.g. ELISA, FACS, IP).

## Data availability

The data referenced by this article are under copyright with the following copyright statement: Copyright: © 2018 Gabay-Maskit S et al.

Data associated with the article are available under the terms of the Creative Commons Zero "No rights reserved" data waiver (CC0 1.0 Public domain dedication). 

**Dataset 1:** Raw images of all western blots included in figures presented. 10.5256/f1000research.13396.d212121 ^9^
