# Supplementary material for: Validation of a yeast malate dehydrogenase 2 (Mdh2) antibody tested for use in western blots
Source: F1000Res. 2018 Jul 30;7:130. Originally published 2018 Jan 31. [Version 2] doi: 10.12688/f1000research.13396.2 (PMC5840644; doi:10.12688/f1000research.13396.2)

Figure 3

Anti-Mdh2 peptide 3

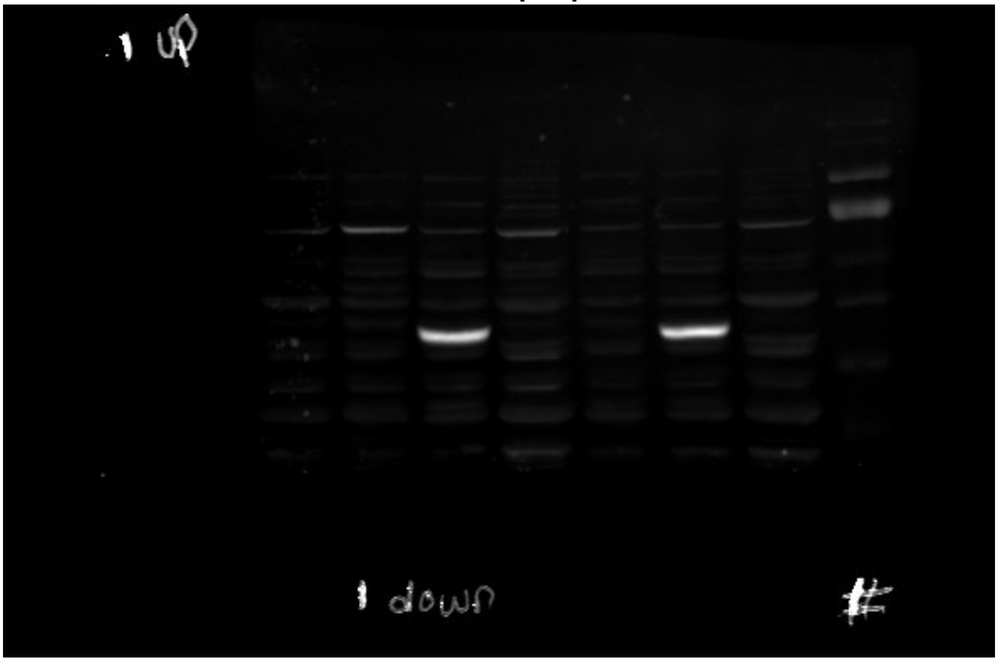

Anti-mCherry and ladder

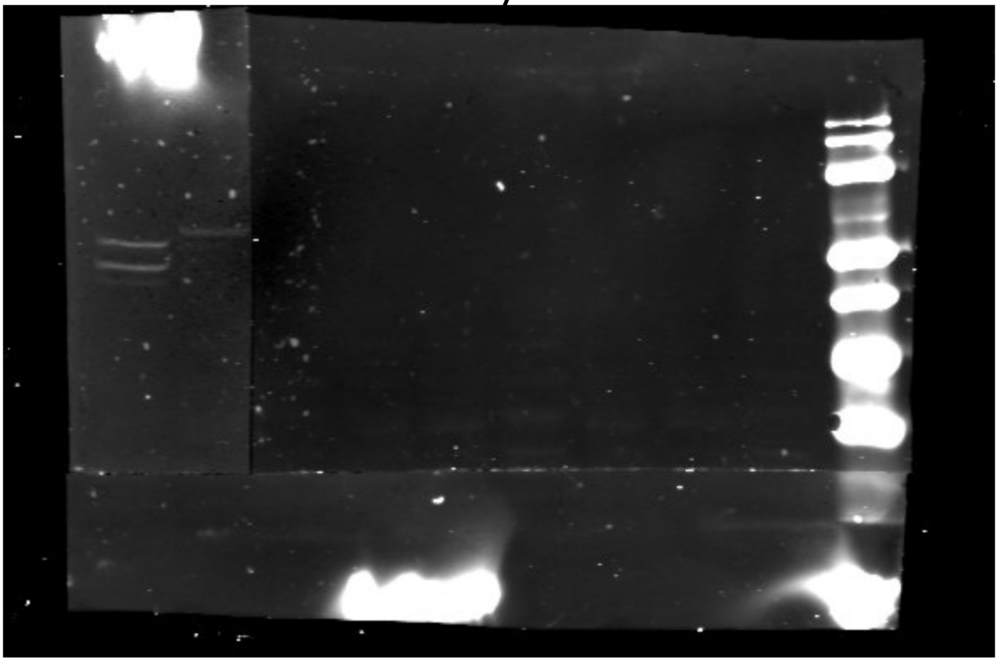

Anti-actin in LAS4000

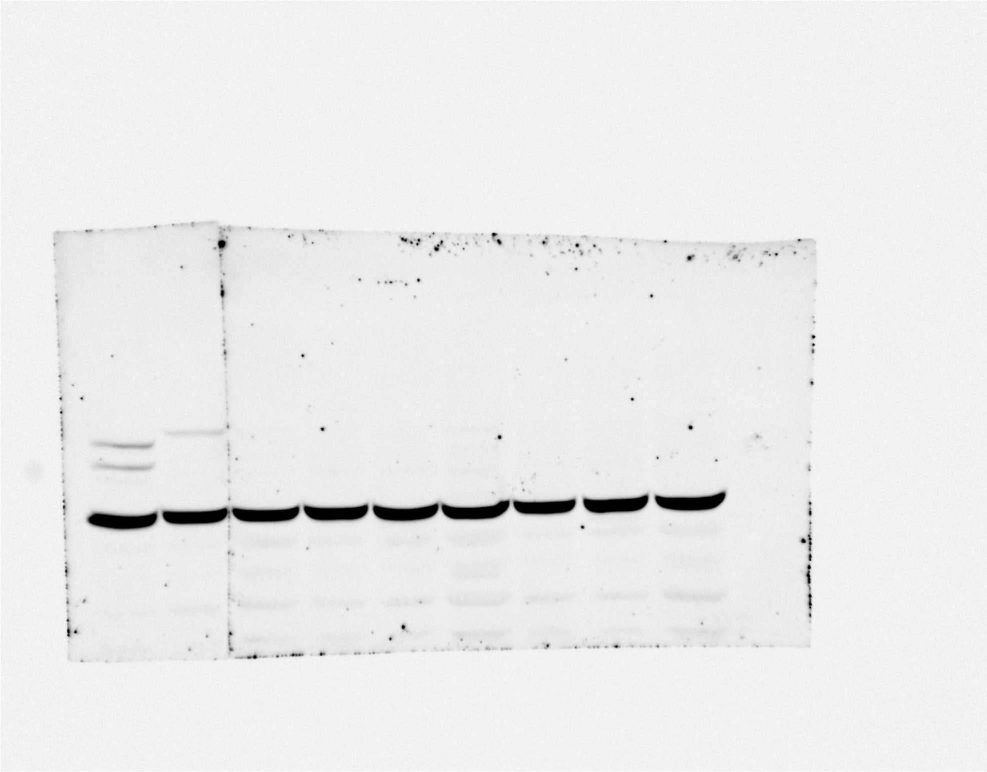

Supplement: Raw images of all western blots included in figures presented [file f1000research-7-17154-s0000.tgz › e908a4d4-5141-441c-8180-b8397048e467_dataset_1_version_2.zip!/dataset 1/Figure_3_raw_not_cropped.pdf]
