# Supplementary material for: Validation of a yeast malate dehydrogenase 2 (Mdh2) antibody tested for use in western blots
Source: F1000Res. 2018 Jul 30;7:130. Originally published 2018 Jan 31. [Version 2] doi: 10.12688/f1000research.13396.2 (PMC5840644; doi:10.12688/f1000research.13396.2)
Supplement: Peer review history

# Peer review: Referee response for version 1

**DOI:** 10.5256/f1000research.14544.r31221

**Published:** 2018-03-06

**Authors:** Ewald H. Hettema

**Affiliations:** 1 Department of Molecular Biology and Biotechnology, University of Sheffield, Sheffield, UK

The title of this manuscript describes exactly its contents. The validation of a S. cerevisiae malate dehydrogenase 2, Mdh2, antibody for use in western blots.

 The study is informative and complete and clearly shows that, of the three antisera tested, the third antiserum is of highest specificity. In total lysates, no cross reactivity is found to related proteins Mdh1 and Mdh3. Conditions of sample preparation and western blot analysis are well described and should ensure for reproducibility. The only thing that could further enhance reproducibility is information on how much protein or culture OD600 equivalents were loaded per lane.

I have read this submission. I believe that I have an appropriate level of expertise to confirm that it is of an acceptable scientific standard.

# Peer review: Referee response for version 1

**DOI:** 10.5256/f1000research.14544.r30437

**Published:** 2018-02-15

**Authors:** Andreas Hartig

**Affiliations:** 1 Department of Biochemistry and Cell Biology, University of Vienna, Vienna, Austria

As an antibody validation study the manuscript presented is complete, sufficient controls are included demonstrating the specificity of the antibodies for the Mdh2-protein with Ab3 sticking out as the best one because of low background. In figures 3 and 4 an unspecific band at the same size as the fusion proteins can be seen in many lanes, which is unfortunate raising doubts whether the Mdh2-mCherry or the GFP-Mdh2 are really specifically recognized. On the other hand, the native Mdh2-band at 41kD is missing in these lanes as expected increasing confidence into the specificity of the antibodies.

 Since Mdh2p is the isoform involved in the glyoxylate cycle it can be expected that this protein will be well expressed under conditions requiring this metabolic pathway, e.g. on ethanol, acetate or oleate. From the data presented in this manuscript it is not possible to conclude that “the upregulation of Mdh2 in cells grown on oleic acid is due to removal of the glucose repression and not because of an oleic acid-specific up-regulation”. For such a statement one would need to compare and present quantitative expression data from cells grown on non-repressing carbon sources such as ethanol and on oleate. It may well be that the expression of Mdh2 is derepressed on ethanol and further induced when grown on oleate. Since this is neither essential nor the primary subject of the antibody validation study I would suggest removing this sentence from the final version of the manuscript.

 I did have troubles in assessing the original data of the Westernblots, but this might be due to the old version of photoshop I’m using on my Computer.

I have read this submission. I believe that I have an appropriate level of expertise to confirm that it is of an acceptable scientific standard.
